# Clinical outcome analysis of patients with autism spectrum disorder: analysis from the UK Medical Cannabis Registry

**DOI:** 10.1177/20451253221116240

**Published:** 2022-09-20

**Authors:** Simon Erridge, Jess Kerr-Gaffney, Carl Holvey, Ross Coomber, Daniela A. Riano Barros, Urmila Bhoskar, Gracia Mwimba, Kavita Praveen, Chris Symeon, Simmi Sachdeva-Mohan, Mikael H. Sodergren, James J. Rucker

**Affiliations:** Imperial College Medical Cannabis Research Group, Department of Surgery and Cancer, Imperial College London, London, UK; Sapphire Medical Clinics, London, UK; Department of Psychological Medicine, Institute of Psychiatry, Psychology & Neuroscience, King’s College London, London, UK; Sapphire Medical Clinics, London, UK; Sapphire Medical Clinics, London, UK; St George’s Hospital NHS Trust, London, UK; Sapphire Medical Clinics, London, UK; Bethlem Royal Hospital, South London and Maudsley NHS Foundation Trust, London, UK; Sapphire Medical Clinics, London, UK; Sapphire Medical Clinics, London, UK; Sapphire Medical Clinics, London, UK; Sapphire Medical Clinics, London, UK; Bethlem Royal Hospital, South London and Maudsley NHS Foundation Trust, London, UK; Sapphire Medical Clinics, London, UK; Imperial College Medical Cannabis Research Group, Department of Surgery and Cancer, Imperial College London, London, UK; Sapphire Medical Clinics, London, UK; Sapphire Medical Clinics, London, UK; Bethlem Royal Hospital, South London and Maudsley NHS Foundation Trust, London, UK; Department of Psychological Medicine, Institute of Psychiatry, Psychology & Neuroscience, King’s College London, 16 De Crespigny Park, London SE5 8AF, UK

**Keywords:** autism spectrum disorder, cannabidiol, cannabis, psychiatry, tetrahydrocannabinol

## Abstract

**Introduction:**

Cannabis-based medicinal products (CBMPs) have been identified as a promising novel therapeutic for symptoms and comorbidities related to autism spectrum disorder (ASD). However, there is a paucity of clinical evidence of their efficacy and safety. Objective: This case series aims to assess changes to health-related quality of life and the incidence of adverse events in patients treated with CBMPs for associated symptoms of ASD enrolled on the UK Medical Cannabis Registry (UKMCR).

**Methods:**

Patients treated with CBMPs for ASD-related symptoms for a minimum of 1 month were identified from the UKMCR. Primary outcomes were changes in validated patient-reported outcome measures [Generalised Anxiety Disorder-7 (GAD-7), Single-Item Sleep Quality Scale (SQS), 5-level version of the EQ-5D (EQ-5D-5L) index values] at 1, 3 and 6 months compared with baseline. Adverse events were recorded and analysed. Statistical significance was determined by *p* < 0.050.

**Results:**

Seventy-four patients with ASD were included in the analysis. The mean age of participants was 32.7 (±11.6) years. There were significant improvements in general health-related quality of life and sleep as assessed by the EQ-5D-5L, SQS and GAD-7 at 1 and 3 months, with sustained changes in EQ-5D-5L and SQS at 6 months (*p* < 0.010). There were 180 (243.2%) adverse events reported by 14 (18.9%) participants. If present, adverse events were commonly mild (*n* = 58; 78.4%) or moderate (*n* = 81; 109.5%), rather than severe (*n* = 41; 55.4%).

**Conclusion:**

This study demonstrated an associated improvement in general health-related quality of life, and anxiety- and sleep-specific symptoms following initiation of treatment with CBMPs in patients with ASD. These findings, while promising, are limited by the confines of the study which lacks a control arm and is subject to attrition bias. Therefore, further evaluation is required with randomised controlled trials.

## Introduction

Autism spectrum disorder (ASD) is a heterogeneous pervasive developmental disorder defined according to deficits in social communication and interaction, in addition to the presence of restricted, repetitive behaviours.^1^ ASD is estimated to affect 1 in 132 individuals globally, and its incidence is increasing.^2^ Individuals with ASD are affected by a higher incidence of comorbid irritability, challenging behaviours, self-injury and psychiatric conditions.^3,4^ Challenging behaviours, such as irritability, destructiveness, aggression and hyperactivity, are estimated to affect anywhere between 56% and 94% of children with a diagnosis of ASD.^3^ These behaviours, except for hyperactivity, have been observed as persisting into adulthood.^3^ ASD is also associated with a higher incidence of physical comorbidities and sleep disturbance in adults.^5–7^ Consequently, ASD has been associated with reduced quality of life for both adult and paediatric patients.^4,8^

Management of the core symptoms of ASD is predominantly via a psychological approach.^9^ However, there is paucity of high-quality research to delineate the effectiveness of different psychological approaches.^4^ At present, there are no established pharmacological treatments for the core symptoms of ASD. Medications, such as monoamine reuptake inhibitors, have been used in the treatment of comorbid psychiatric illnesses, although the efficacy in people with ASD compared with the general population is not well-known.^4^ Atypical neuroleptics, such as risperidone and aripiprazole, have demonstrated efficacy in treating irritability and aggression in children and adolescents.^10^ However, these medications are poorly tolerated due to their side effect profile,^10^ and their efficacy in an adult population with ASD is again unknown. Consequently, there is an unmet clinical need to identify novel therapeutics for the core symptoms of ASD, other associated symptoms or comorbid diagnoses.

The endocannabinoid system has been implicated in the pathophysiology of ASD, as well as is a potential pharmaceutical target. Cannabinoid type 1 receptors (CB1Rs) are predominantly located in the central nervous system and at increased density within the basal ganglia, hippocampus and cerebellum.^11^ Moreover, CB1Rs are principally expressed at pre-synaptic terminals of γ-aminobutyric acid (GABA)ergic and glutaminergic neurons.^12^ Endogenous agonists of CB1Rs, such as anandamide, cause inhibition of either GABA or glutamate synaptic release.^12^ Subsequently, the endocannabinoid system has been implicated in the regulation of anxiety, mood, motor coordination and social behaviour.^11–13^ Children with ASD have been found to have lower circulating levels of anandamide compared with the general population.^14^

Cannabidiol (CBD), a major phytocannabinoid, is an inhibitor of anandamide reuptake and breakdown, a negative allosteric modulator of CB1Rs and is an agonist of 5-HT1a serotonin receptors.^12^ (−)-trans-Δ^9^-tetrahydrocannabinol (Δ^9^-THC), another active pharmaceutical ingredient of the cannabis plant, is an agonist of CB1Rs.^12^ Cannabis-based medicinal products (CBMPs), containing these phytocannabinoids, have therefore been highlighted as a potential class of medications for utilisation across the broad potential symptom profile of ASD. In the United Kingdom, CBMPs may be considered for these symptoms if licensed treatments have failed to produce a sufficient clinical response or are not tolerated.^15^ In 2019, Schleider *et al.*^16^ published a series of outcomes from 188 children and adolescents treated with CBMPs. In this study they demonstrated an improvement in quality of life, mood, sleep and challenging behaviours. However, they did not utilise any validated measures to assess for symptom prevalence and change over time.^16^ At present, there is a paucity of randomised controlled trials and other high-quality evidence on the efficacy and safety of CBMPs in the treatment of ASD-associated symptoms. Importantly, there are no published clinical studies of the outcomes of adult patients treated with CBMPs. Herein, the primary aim of this study is to report the general health-related quality of life outcomes and adverse event incidence of patients prescribed CBMPs for ASD enrolled on the UK Medical Cannabis Registry.

## Methods

### Study design and database

Extraction of an uncontrolled case series of patients from the UK Medical Cannabis Registry of patients treated with CBMPs for ASD was performed. The UK Medical Cannabis Registry is a bespoke prospective registry, which has collected outcomes on patients prescribed CBMPs in the United Kingdom and Channel Islands since 2019.^17^ It is the largest registry of its kind in the United Kingdom, with data on more than 3500 pseudonymised patients treated with CBMPs. Participants are recruited from Sapphire Medical Clinics, a specialist private clinic. All patients completed formal, written consent prior to enrolment in the registry.

The study was reported in line with the STROBE (Strengthening the Reporting of Observational Studies in Epidemiology) statement for reporting observational studies.^18^

### Patient and data selection

Inclusion criteria for this study were patients who were receiving treatment with CBMPs where ASD was the primary condition for which treatment was commenced. Patients treated for other conditions, where ASD was not the primary indication, were excluded. Participants who had not completed baseline patient-reported outcome measures (PROMs) or who had received treatment for less than 1 month were also excluded from analysis.

The clinicopathological characteristics of study participants were recorded by clinicians and extracted at baseline, including age, gender, body mass index (BMI) and comorbidities. The Charlson Comorbidity Index was calculated to allow comparison of comorbidity against other epidemiological studies.^19^ This is calculated using baseline age, and the presence or absence of the following comorbidities: myocardial infarction, congestive heart failure, peripheral vascular disease, cerebrovascular accident/transient ischaemic attack, dementia, chronic obstructive pulmonary disease, connective tissue disease, peptic ulcer disease, liver disease, diabetes mellitus, hemiplegia, chronic kidney disease, solid tumour, leukaemia, lymphoma and acquired immune deficiency syndrome.^19^ A higher index score is associated with an increased incidence of mortality at 1 and 10 years.^19^

Drug and alcohol data were collated in line with previously published research from our group.^20–22^ Concurrent medications taken at baseline by a patient and any changes to medications were self-recorded by patients via a remote data collection platform or by clinicians during routine follow-up. These were mapped to SNOMED CT codes to improve the accuracy of reporting and identification.

CBMPs were recorded throughout treatment. All prescriptions were for CBMPs that adhered to Good Manufacturing Practice. Patients could be prescribed sublingual/oral or vapourised preparations according to clinical requirements. In accordance with UK guidance, CBMPs were only initiated by consultant physicians in their clinical area of interest.^15^ The doses of major cannabinoids, CBD and Δ^9^-THC, prescribed at the time of data extraction in milligrams (mg) per day were extracted, in addition to specific CBMP prescribed.

The primary outcomes were changes in the Generalised Anxiety Disorder Scale (GAD-7), 5-level version of the EQ-5D (EQ-5D-5L) index value and Sleep Quality Scale (SQS) from baseline to 1, 3 and 6 months. These were assessed in line with previously described methodology from our group.^17,20,21^ The GAD-7 is a validated scale to assess the severity of generalised anxiety symptoms. Scores of ⩾5, ⩾10 and ⩾15 represent mild, moderate and severe anxiety levels, respectively.^23^ The EQ-5D-5L index value is a measure of health-related quality of life where a score of 1 represents an ideal health state, while a score less than 0 is equivalent to being worse than death.^24^ The SQS is a single-item scale from 0 to 10 of sleep quality whereby 0 is ‘terrible’ sleep quality and 10 is ‘excellent’.^25^ The Patient Global Impression of Change (PGIC) was also reported at 1, 3 and 6 months. The PGIC is a 7-point numerical rating scale where participants self-rate the change in activity limitations, symptoms, emotions and overall quality of life since starting treatment. A score of 1 represents ‘no change’, while a score of 7 represents ‘a considerable improvement’.^26^

Secondary outcome measures were the incidence of adverse events recorded utilising the Common Terminology Criteria for Adverse Events v4.0.^27^ Adverse events were self-reported by patients when completing PROMs or completed by clinicians during routine follow-up.

### Statistical analysis

Data about patient clinicopathological characteristics, drug and alcohol consumption, CBMP prescriptions and adverse events were analysed using descriptive statistics. The Shapiro–Wilk test was used to assess the distribution of studied data. Unless otherwise stated, parametric data were presented as mean ± standard deviation (SD), while non-parametric data were presented as median [interquartile range (IQR)]. Frequencies were presented as *n* (%). Analysis of PROMs was performed using a paired *t*-test for parametric data or the Wilcoxon signed-rank test for non-parametric data. Due to the number of analyses performed on the PROMs, a Benjamini–Hochberg procedure was performed to control for the false discovery rate. Statistical significance was defined as *p*-value <0.050. All analyses were performed in Statistical Package for Social Sciences (SPSS) (IBM Statistics version 27.0.0.0 SPSS Inc).

## Results

Seventy-four patients were included in the analysis. The median follow-up was 204.0 (range: 34.0–557.0) days. Completion of PROMs varied at 1 (*n* = 60; 81.1%), 3 (*n* = 49; 66.2%) and 6 (*n* = 31; 41.9%) months. The mean age of the participants was 32.7 (±11.6) years. Fifty-three (71.6%) participants were male. The mean BMI was 26.3 (±6.4) kg/m^2^. Table 1 details the full clinicopathological characteristics of study participants at baseline.

**Table 1.** Clinicopathological characteristics of study participants at baseline.

Baseline characteristics	*n* (%) / Mean ± SD / Median [IQR]
Gender	
Female	21 (28.4%)
Male	53 (71.6%)
Age	32.7 ± 11.6
Body mass index (kg/m^2^)	26.3 ± 6.4
Occupation	
Unemployed	46 (62.2%)
Undisclosed	8 (10.8%)
Professional	7 (9.5%)
Service and sales workers	5 (6.8%)
Elementary occupations	3 (4.1%)
Other occupations	2 (2.7%)
Clerical support workers	1 (1.4%)
Managers	1 (1.4%)
Technicians and associate professionals	1 (1.4%)
Charlson Comorbidity Index	0.0 [0.0–0.0]
Anxiety/depression	51 (68.9%)
Arthritis	0 (0.0%)
Endocrine dysfunction	2 (2.7%)
Epilepsy	3 (4.1%)
Hypertension	1 (1.4%)
Venous thromboembolism	0 (0.0%)

IQR, interquartile range.

The majority of patients were regular cannabis consumers at baseline (*n* = 50; 67.6%) (Table 2). The median lifetime exposure to cannabis was 8.0 [IQR: 2.0–20.0] gram years. The median weekly alcohol consumption of the cohort was 0.0 [IQR: 0.0–1.5] units.

**Table 2.** Tobacco, alcohol and cannabis exposure of patients at baseline.

Tobacco, alcohol and cannabis status	*n* (%)/ median [IQR]
Cannabis status	
Cannabis naïve	14 (19.9%)
Ex-user	10 (13.5%)
Current user	50 (67.6%)
Cannabis use, gram years	8.0 [2.0–20.0]
Tobacco status	
Non-smoker	26 (35.1%)
Ex-smoker	24 (32.4%)
Current smoker	24 (32.4%)
Tobacco pack years	5.0 [2.0–15.0]
Weekly alcohol consumption, units	0.0 [0.0–1.5]

IQR, interquartile range.

### Cannabis-based medicinal product dosing

Thirty-six (48.6%) participants were prescribed dried flower CBMPs only, 16 (21.6%) participants were prescribed oral/sublingual preparations and 22 (29.7%) were prescribed both CBMPs. The median prescribed CBD dose per day at the date of extraction was 10.0 [IQR: 4.0–100.0] mg, while the median Δ^9^-THC dose was 112.5 [IQR: 91.3–202.5] mg per day.

### Patient-reported outcome measures

Table 3 outlines in full paired baseline and follow-up results of PROMs up to 6 months. There were significant improvements in general health-related quality of life and sleep, as assessed by the EQ-5D-5L and SQS, respectively, at 1, 3 and 6 months (*p* < 0.010). There were reductions in anxiety severity, as measured by the GAD-7 scale, at 1 and 3 months (*p* < 0.001). However, there was no change at 6 months (*p* = 0.102). The median PGIC value was 6.0 at 1, 3 and 6 months.

**Table 3.** Paired baseline and follow-up patient-reported outcome measures.

	*n*	Baseline score	Follow-up score	*p*-value
GAD-7				
1 month	59	15.0 [8.0–18.0]	7.0 [5.0–12.8]	<0.001
3 months	48	13.0 [6.8–18.0]	6.0 [4.0–12.5]	<0.001
6 months	30	11.5 [5.0–18.0]	7.0 [5.0–13.0]	0.102
SQS				
1 month	59	3.0 [2.0–6.0]	6.0 [3.0–8.0]	<0.001
3 months	48	4.0 [2.0–6.0]	6.0 [4.0–8.0]	<0.001
6 months	30	4.0 [1.8–7.0]	5.5 [3.8–8.0]	0.005
EQ-5D-5L Mobility				
1 month	60	1.0 [1.0–2.8]	1.0 [1.0–2.0]	0.581
3 months	49	1.0 [1.0–3.0]	1.0 [1.0–2.0]	0.027
6 months	31	1.0 [1.0–3.0]	1.0 [1.0–2.0]	0.183
EQ-5D-5L Self-Care				
1 month	60	2.0 [1.0–3.0]	1.5 [1.0–2.8]	0.131
3 months	49	2.0 [1.0–3.0]	1.0 [1.0–2.5]	0.125
6 months	31	1.0 [1.0–2.0]	1.0 [1.0–2.0]	0.441
EQ-5D-5L Usual Activities				
1 month	60	3.0 [2.0–4.0]	2.0 [1.0–3.0]	0.002
3 months	49	3.0 [2.0–4.0]	2.0 [1.0–3.0]	0.002
6 months	31	3.0 [2.0–3.0]	2.0 [1.0–3.0]	0.029
EQ-5D-5L Pain and Discomfort				
1 month	60	2.0 [1.0–3.0]	2.0 [1.0–3.0]	0.005
3 months	49	2.0 [1.0–3.0]	2.0 [1.0–3.0]	0.003
6 months	31	2.0 [1.0–3.0]	2.0 [1.0–3.0]	0.124
EQ-5D-5L Anxiety and Depression				
1 month	60	3.5 [2.0–4.0]	3.0 [2.0–4.0]	<0.001
3 months	49	3.0 [2.0–4.0]	3.0 [2.0–3.0]	0.003
6 months	31	3.0 [2.0–4.0]	3.0 [2.0–3.0]	0.425
EQ-5D-5L Index Value				
1 month	60	0.44 ± 0.31	0.59 ± 0.29	<0.001
3 months	49	0.49 ± 0.30	0.66 ± 0.24	<0.001
6 months	31	0.54 ± 0.26	0.63 ± 0.23	0.008
PGIC				
1 month	60	–	6.0 [5.0–6.0]	–
3 months	46	–	6.0 [5.0–7.0]	–
6 months	30	–	6.0 [5.0–6.0]	–

GAD-7, Generalised Anxiety Disorder Scale; PGIC, Patient Global Impression of Change; SQS, Sleep Quality Scale.

### Co-administered medications

The most commonly co-administered class of medications were antidepressants (*n* = 45; 60.8%), antiepileptics (*n* = 7; 9.5%), benzodiazepines (*n* = 12; 16.2%), neuroleptics (*n* = 12; 16.2%) and stimulants (*n* = 6; 8.1%) (Table 4). In all, 33.3% (*n* = 4) and 25.0% (*n* = 3) of patients stopped taking benzodiazepines and neuroleptics, respectively, during treatment with CBMPs (Table 4).

**Table 4.** Changes in co-administered medications throughout treatment with cannabis-based medicinal products to end of follow-up.

Medication	Total	Stopped taking	Reduced dose	No change	Increased dose	New medication
Antidepressants, *n* (%)	45	5 (11.1)	0 (0.0)	37 (82.2)	0 (0.0)	3 (6.7)
Antiepileptics, *n* (%)	7	1 (14.3)	0 (0.0)	6 (85.7)	0 (0.0)	0 (0.0)
Benzodiazepines, *n* (%)	12	4 (33.3)	0 (0.0)	8 (66.7)	0 (0.0)	0 (0.0)
Neuroleptics, *n* (%)	12	3 (25.0)	0 (0.0)	9 (75.0)	0 (0.0)	0 (0.0)
Stimulants, *n* (%)	6	0 (0.0)	0 (0.0)	6 (100.0)	0 (0.0)	0 (0.0)

### Adverse events

Fourteen (18.9%) participants reported 180 (243.2%) total adverse events. If present, adverse events were commonly mild (*n* = 58; 78.4%) or moderate (*n* = 81; 109.5%), rather than severe (*n* = 41; 55.4%). There were no (0.0%) life-threatening or disabling adverse events in this group (Table 5).

**Table 5.** Adverse events reported by participants.

Adverse event	Mild	Moderate	Severe	Total
Abdominal pain (upper)	1	1	1	3 (4.1%)
Agitation	–	1	2	3 (4.1%)
Amnesia	1	3	1	5 (6.8%)
Anorexia	–	3	3	6 (8.1%)
Anxiety	–	–	1	1 (1.4%)
Ataxia	3	1	1	5 (6.8%)
Blurred vision	1	3	2	6 (8.1%)
Cognitive disturbance	1	2	2	5 (6.8%)
Concentration impairment	5	4	1	10 (13.5%)
Confusion	–	–	2	2 (2.7%)
Constipation	–	1	–	1 (1.4%)
Delirium	1	–	1	2 (2.7%)
Depression	–	–	4	4 (5.4%)
Diarrhoea	–	1	–	1 (1.4%)
Dizziness	2	2	1	5 (6.8%)
Dry mouth	10	1	–	11 (14.9%)
Dysgeusia	1	1	2	4 (5.4%)
Dyspepsia	4	2	–	6 (8.1%)
Fall	–	2	–	2 (2.7%)
Fatigue	3	3	4	10 (13.5%)
Fasciculations	–	1	–	1 (1.4%)
Generalised muscle weakness	2	2	1	5 (6.8%)
Headache	–	7	1	8 (10.8%)
Increased seizure frequency	–	1	2	3 (4.1%)
Insomnia	2	7	4	13 (17.6%)
Lethargy	3	7	1	11 (14.9%)
Nausea	4	2	–	6 (8.1%)
Pharyngitis	–	3	–	3 (4.1%)
Pyrexia	–	1	–	1 (1.4%)
Rash	2	–	–	2 (2.7%)
Somnolence	–	9	–	9 (12.2%)
Spasticity	2	3	1	6 (8.1%)
Tremor	5	–	2	7 (9.5%)
Vertigo	2	1	1	4 (5.4%)
Vomiting	2	–	–	2 (2.7%)
Weight loss	1	3	–	4 (5.4%)
Upper respiratory tract infection	–	3	–	3 (4.1%)
Total	58 (78.4%)	81 (109.5%)	41 (55.4%)	180 (243.2%)

## Discussion

The results of this study are the first published observational data of CBMP therapy focused on adult participants with ASD. They demonstrate an associated improvement in general health-related quality of life, sleep and anxiety in patients with ASD after commencement of therapy with CBMPs. There was also a 33.3% and 25.0% reduction in the concomitant prescribing of benzodiazepines and neuroleptics, respectively, within this cohort. Adverse events were experienced by 18.9% of the cohort with a total adverse event incidence of 243.2%. However, the findings must be interpreted cautiously due to the limitations of study design.

Improvements in health-related quality of life are supported by previous evaluations of CBMPs in the setting of ASD. A systematic review by Fusar-Poli *et al.*^28^ identified that the majority of studies have found improvements in problem behaviours, hyperactivity, parental stress and other studied outcomes. However, the present study is the first to report these findings in an adult population. Previous studies have only reported outcomes for patients aged up to 22 years, while the mean age of the present case series was 32.7 years. However, adult patients with ASD have reported using medical cannabis and illicitly obtained cannabis for mental health symptoms or challenging behaviours.^29,30^ The finding of improved health-related quality of life is, however, supported by findings from other studies of UK patients treated with CBMPs. A study of 312 patients with all conditions from the UK Medical Cannabis Registry published by our group, similarly, demonstrated improved health-related quality of life as measured by the EQ-5D-5L index value.^22^ Moreover, similar results were published in a cohort of patients with generalised anxiety disorder.^31^

Adults with ASD have a higher prevalence of comorbid sleep disorders.^5^ The present study found an associated improvement in self-reported sleep quality among participants at up to 6 months. The effect of CBMPs on sleep is disputed; however, the endocannabinoid system has been implicated in the regulation of the sleep–wake cycle.^32^ A recent randomised controlled trial of a CBMP containing CBD, Δ^9^-THC and cannabigerol found improvements in reducing sleep-onset latency and waking after sleep onset.^33^ However, this study only lasted 2 weeks and was limited to a small sample size. Other studies have raised concerns with respect to developing tolerance to the sleep-promoting effects of CBMPs with longitudinal use.^34^ In addition, insomnia was the most frequently reported adverse event in this study, highlighting that the benefits of CBMPs on sleep may also be negative in certain patients. A long-term pharmacovigilance strategy, such as the UK Medical Cannabis Registry, shall be necessary for continued evaluation of the benefits and risks of long-term prescribing of CBMPs.

The present study demonstrated statistically significant improvements in generalised anxiety symptoms at 1 and 3 months; however, the same findings were not present at 6 months. This divergence at 6 months appears to be secondary to a limitation in study design, whereby there is a reduction in the number of patients followed up to 6 months. Therefore, there is a reduction in sample size, such that despite a trend towards improved generalised anxiety symptoms at 6 months, the finding is not significant. In addition, the baseline values indicate a lower severity of anxiety. The reason for incomplete follow-up is not recorded in the UK Medical Cannabis Registry; however, this may be secondary to those patients with the most severe anxiety symptoms at baseline not continuing treatment up to 6 months due to being unable to tolerate therapy, insufficient clinical response or a non-clinical reason such as cost of treatment. Previous evaluations of the children and young adult patients with ASD have similarly found associated improvements in anxiety outcomes.^28^

There was a reduction in concomitant medication use during treatment. Benzodiazepines are not recommended for the long-term treatment of aggression or irritability in ASD as they can induce tolerance and are associated with a high incidence of adverse events.^35^ In all, 33.3% of participants stopped taking benzodiazepines during treatment. Atypical neuroleptics risperidone and aripiprazole are licensed for the treatment of irritability associated with ASD.^35^ However, long-term use is associated with metabolic syndrome. In this study, there were reductions in neuroleptic (25.0%) prescribing. Observational studies in children have also shown that administration of CBMPs can lead to a reduction in other medications.^28^ The long-term efficacy and safety of this approach, however, need evaluation in long-term observational series and randomised controlled trials.

Adverse events were reported by 18.9% of participants, with a total incidence of 243.2%. This incidence is higher than reported in previous observational series.^36–38^ The reason for this is likely due to the methodology for collecting adverse events. First, patients are prompted to report adverse events remotely alongside PROMs at 1, 3 and 6 months. In addition, they may report adverse events during a clinical encounter. This methodology therefore represents one of the most sophisticated pharmacovigilance strategies for CBMPs globally at present. Similar to other studies, CBMPs were well tolerated by most patients as 81.1% of patients did not experience any adverse events.^36–38^ Moreover, there were no life-threatening or disabling adverse events, while most adverse events were mild (78.4%) or moderate (109.5%).

Despite being the first published experience of clinical outcomes for adult patients with ASD prescribed CBMPs, there are limitations to the present study design. There is no control group for comparison, and therefore, it is not possible to determine whether the associated changes are caused by CBMPs and not due to another cause, such as regression to the mean. The study participants are all recruited from a private medical clinic and therefore may not be representative of the general population. However, 62.2% of participants were unemployed, indicating that the cost of treatment was not wholly prohibitive to starting treatment. Moreover, paying for treatment may enhance the placebo effect, which is already enhanced in CBMPs due to the associated aroma, and psychoactive and vasoactive effects.^39^ Finally, attrition bias is likely due to loss to follow-up, which subsequently reduces the internal validity of the study. To ensure that changes in individuals were measured accurately, changes were compared with baseline rather than previous follow-up period.

## Conclusion

In this first published experience of clinical outcomes in adult patients with ASD treated with CBMPs, there were associated improvements in general health-related quality of life, in addition to sleep- and anxiety-specific outcomes. Moreover, there was a reduction in the administration of concomitant medications, some of which are associated with serious adverse events with long-term use. CBMPs were well tolerated by the majority (81.1%) of patients. These results must be interpreted within the context of the limitations of study design, and causation cannot be determined. However, it provides scientific justification for further evaluation within the context of randomised controlled trials while also providing guidance for clinical practice in the interim.

## Declarations

**Ethics approval and consent to participate:** As a registry study, and as advised by the NHS Health Research Authority and Research Ethics Committee, formal ethical approval was not required. Informed consent was provided by all participants prior to enrolment in the registry.

**Consent for publication:** Not applicable.

**Availability of data and material:** The data that support the findings of this study are available on request from the corresponding author. The data are not publicly available due to privacy or ethical restrictions. The raw data that belonged to the present study cannot be made publicly available because the disclosure of personal data, albeit pseudonymised, was not included in the research protocol of the present study.
